# Calcineurin B-like Protein CBL10 Directly Interacts with TOC34 (Translocon of the Outer Membrane of the Chloroplasts) and Decreases Its GTPase Activity in Arabidopsis

**DOI:** 10.3389/fpls.2016.01911

**Published:** 2016-12-15

**Authors:** Joo Hyuk Cho, Jeong Hwan Lee, Yoon Kook Park, Mi Na Choi, Kyung-Nam Kim

**Affiliations:** Department of Molecular Biology, PERI, Sejong UniversitySeoul, South Korea

**Keywords:** calcium signature, CBL10, CIPK, GTPase activity, TOC34

## Abstract

As calcium sensor relays in plants, calcineurin B-like (CBL) proteins provide an important contribution to decoding Ca^2+^ signatures elicited by a variety of abiotic stresses. Currently, it is well known that CBLs perceive and transmit the Ca^2+^ signals mainly to a group of serine/threonine protein kinases called CBL-interacting protein kinases (CIPKs). In this study, we report that the CBL10 member of this family has a novel interaction partner besides the CIPK proteins. Yeast two-hybrid screening with CBL10 as bait identified an Arabidopsis cDNA clone encoding a TOC34 protein, which is a member of the TOC (Translocon of the Outer membrane of the Chloroplasts) complex and possesses the GTPase activity. Further analyses showed that in addition to CBL10, CBL7 also interacts with TOC34 at much lower strength in the yeast two-hybrid system. However, the rest of the CBL family members failed to interact with TOC34. Bimolecular fluorescence complementation (BiFC) analysis verified that the CBL10-TOC34 interaction occurs at the outer membrane of chloroplasts *in vivo*. In addition, we also demonstrated that CBL10 physically associates with TOC34 *in vitro*, resulting in a significant decrease in the GTPase activity of the TOC34 protein. Taken together, our findings clearly indicate that a member of the CBL family, CBL10, can modulate not only the CIPK members but also TOC34, allowing the CBL family to relay the Ca^2+^ signals in more diverse ways than currently known.

## Introduction

Plants as sessile organisms need to adapt to environmental changes by regulating their cellular and physiological status. It is well known that these adaptation processes are often preceded by transient increases of free Ca^2+^ concentrations in the cytosol of plant cells (White and Broadley, 2003). In fact, Ca^2+^ serves as a versatile second messenger when plants respond to a wide range of environmental stimuli such as biotic and abiotic stresses (McAinsh and Pittman, 2009). Each of these stresses can induce a unique Ca^2+^ signature, which is comprised of not only the magnitude but also the temporal and spatial parameters including frequency, duration, and subcellular localization of the Ca^2+^ oscillations (Evans et al., 2001; Rudd and Franklin-Tong, 2001; Sanders et al., 2002).

In order to decode these distinct Ca^2+^ signals and give rise to specific responses, plants should be equipped with many Ca^2+^ sensors harboring particular characteristics in terms of Ca^2+^-binding affinity, expression patterns, subcellular localization, and interaction partners. Indeed, plants possess a number of distinct Ca^2+^ sensor proteins, which contain the canonical EF-hand Ca^2+^-binding motifs. These Ca^2+^-binding proteins are largely classified into several families; calmodulin (CaM), CaM-like protein (CML), Ca^2+^-dependent protein kinase (CDPK), caleosin, and calcineurin B-like protein (CBL) (Batistic and Kudla, 2012; Kim, 2013; Shen et al., 2014). Except for CaM that is probably best known and highly conserved in all eukaryotes, the other families including CML, CDPK, and CBL are unique to plants (Carafoli, 2002; Batistic and Kudla, 2009). In the case of caleosin, it is found in both plants and fungi. Meanwhile, it is interesting to note that plants also have another type of Ca^2+^-binding proteins that do not depend on the EF-hand motif (e.g., C2-domain containing proteins), although their biological function and regulatory mechanisms are not well understood (Reddy and Reddy, 2004).

These plant Ca^2+^-binding proteins can be group into sensor responders and sensor relays according to the mode of decoding Ca^2+^ signals (Sanders et al., 2002). Sensor responders include proteins with enzymatic activity modulated by an intramolecular Ca^2+^-binding domain. Therefore, CDPKs belong to sensor responders, because they carry a kinase domain (responder) at the N-terminal end and a regulatory Ca^2+^-binding domain (sensor) at the C-terminus. Eventually, they transduce the information encoded in Ca^2+^ signatures into phosphorylation events of specific downstream target proteins. On the contrary, sensor relays lacking enzyme activity associate with and regulate other proteins in a Ca^2+^-dependent manner. The well-known examples of sensor relays are CaM and CML family members, which undergo conformational changes upon Ca^2+^ binding and target various proteins such as kinase, channel proteins, metabolic enzymes, transcription factors, and so forth (Kim et al., 2002; Yang and Poovaiah, 2003; Park et al., 2005; Dobney et al., 2009; Oh et al., 2012). Due to this target diversity, the CaM and CML sensor relays can control a variety of cellular and physiological processes in response to diverse Ca^2+^-eliciting stimuli.

Studies with the CBL Ca^2+^-binding proteins revealed them as sensor relays as well because they do not have any enzymatic activity and activate a group of serine/threonine protein kinases called CIPKs (CBL-interacting protein kinases) in a Ca^2+^-dependent manner (Liu and Zhu, 1998; Kudla et al., 1999; Shi et al., 1999). The CIPK proteins are unique to plants in that they contain a distinct regulatory domain at the C-terminus along with the N-terminal kinase domain similar to the yeast SNF1 protein kinase (Sucrose Non-Fermenting 1) and the Ca^2+^ mammalian AMP-dependent protein kinase (Shi et al., 1999). The CBL members physically interact with the conserved NAF (or FISL) motif in the CIPK C-terminal regulatory domain, which serves as an auto-inhibitory module by blocking the active site of the kinase domain (Guo et al., 2001). Upon interaction with a Ca^2+^-bound CBL partner, CIPKs undergo a conformational change to displace the auto-inhibitory domain, resulting in gaining the phosphorylation activity (Chaves-Sanjuan et al., 2014).

It was firmly believed that CBLs target only the CIPK members, because no other CBL-interaction partners had been reported in spite of extensive investigations carried out by various research groups. As a matter of fact, all the CBL-related reports published over a decade indicated that CBLs associate exclusively with the CIPK members, thereby mediating Ca^2+^ signals elicited by various stimuli including cold, high salinity, low K^+^ concentration, high pH, abscisic acid (ABA), and osmotic stress (reviewed in Kim, 2013). This narrow spectrum of CBL targets was somewhat unexpected as compared with the target diversity exhibited by other sensor relays such as CaMs and CMLs. Recently, however, this belief has been overturned due to our previous two reports, in which we demonstrated that one of the CBL family members, CBL3, specifically interacts with and inhibits the Arabidopsis 5′-methylthioadenosine nucleosidase (AtMTAN) family members in a Ca^2+^-dependent manner (Oh et al., 2008; Ok et al., 2015). These findings raised a possibility that each of the CBL members can have distinct interaction partner proteins besides CIPKs and prompted us to seek out new proteins targeted by the other CBL family members. It is essential to identify all the CBL-interacting partners in order to unravel the Ca^2+^ signaling pathways mediated by the CBL family members in plants.

Therefore, in this study, we performed extensive yeast two-hybrid screening of Arabidopsis cDNA libraries using CBL10 as a bait to isolate an interaction partner(s) which does not belong to the CIPK family. We selected CBL10 among the 10 CBL family members in Arabidopsis, because it interacted with the fewest number of CIPK members in our previous experiments. Through the screening, we identified a novel CBL10 interactor, Translocon of the Outer membrane of the Chloroplasts 34 (TOC34), which act as a GTP-dependent receptor at the chloroplast surface (Andrès et al., 2010). We further demonstrated that CBL10 physically interacts with TOC34 and significantly inhibits its GTPase activity upon Ca^2+^ binding, providing an additional level of complexity for the existing CBL-mediated Ca^2+^-signaling networks. Taken together, our findings clearly suggest that each member of the CBL family can have distinct target proteins along with the CIPK proteins, thereby transmitting Ca^2+^ signals in much more diverse ways to regulate many biochemical and physiological processes in plants.

## Materials and methods

### Yeast two-hybrid screening and assays

The Arabidopsis (*Arabidopsis thaliana*) cDNA expression libraries (CD4-10 and CD4-22) were obtained from the Arabidopsis Biological Resource Center (ABRC) and used in the yeast two-hybrid screening, which was performed basically according to Oh et al. (2008). For yeast two-hybrid assays, full-length open reading frames (ORFs) for genes of interest were amplified from total RNA of Arabidopsis seedlings by reverse transcription (RT)-polymerase chain reaction (PCR) using gene-specific primer sets and subsequently cloned into either the activation domain (pGAD.GH) or the DNA binding domain (pGBT9.BS) vectors. These constructs were then introduced into yeast strain Y190 by the lithium acetate method (Schiestl and Gietz, 1989). Yeast transformants carrying both plasmids were selected on the synthetic medium lacking Leu and Trp (SC-LW) for 3–5 days at 30°C. The yeast cells were subsequently streaked on the synthetic complete medium lacking His, Leu and Trp (SC-HLW) plate to determine the expression of the *HIS3* nutritional reporter gene.

### Plant materials and RNA expression analysis

Arabidopsis [ecotype Columbia (Col-0)] plants were grown in a growth chamber at 23°C under long-day (LD) conditions (16-h-light/8-h-dark cycle) at a light intensity of 120 μmol m^−2^ s^−1^. The total RNA was isolated from a variety of tissues using the plant RNA mini kit (Qiagen, Germany) according to the manufacturer's instructions. RNA quality was determined with a Nanodrop ND-2000 spectrophotometer (Nanodrop Technologies, USA), and only high quality RNA samples (A_260_/A_230_ > 2.0 and A_260_/A_280_ > 1.8) were used for subsequent experiments. To remove possible genomic DNA contamination, RNA samples were treated with DNaseI for 60 min at 37°C. RNA (1 μg) was used for complementary DNA (cDNA) synthesis, in accordance with the manufacturer's instructions (New England Biolabs). Quantitative real-time reverse transcription PCR (qRT-PCR) was carried out using a Rotor-Gene Real-Time Centrifugal DNA Amplification system (Corbett Research). PCR reactions were performed using the QuantiTect SYBR Green PCR Master MIX following the manufacturer's instructions (Qiagen). Data analysis was performed with Rotor-Gene software and relative amounts of mRNA were determined based on the comparative threshold cycle method. The housekeeping gene *Actin2* was used as a reference gene to normalize the relative expression of target genes, according to “The eleven golden rules for quantitative RT-PCR” (Udvardi et al., 2008). All qRT–PCR experiments were carried out in two biological replicates (independently harvested samples) with three technical replicates. Oligonucleotide sequences used for the expression analysis are provided below: CBL10-F (5′-TTCATTGAGCGAGAAGAGGTGCA-3′), CBL10-R (5′- GGAATGCTGTCGTCACAT CCTTT-3′), TOC34-F (5′- TGCTGCAGTTAGTACTTTCCAGTCT-3′), TOC34-R (5′- TAT AGTCATGTTGAGGAGAAATCGT-3′), Actin2-F (5′- TGAGGATATTCAGCCACTTGT CTG-3′), and Actin2-R (5′- GATTGGATACTTCAG AGTGAGGAT-3′).

### Subcellular localization and bimolecular fluorescence complementation (BiFC) analyses

For subcellular localization and BiFC analyses in Arabidopsis protoplasts, different plasmids were transformed into Arabidopsis mesophyll cells by a PEG-mediated transfection procedure (Yoo et al., 2007). After the transfected Arabidopsis protoplasts were incubated at 23°C for 18 h, fluorescence signals were analyzed with a confocal laser scanning microscope (LSM 510 META, Carl Zeiss). For BiFC analysis in tobacco (*Nicotiana benthamiana*) leaves, the *Agrobacterium tumefaciens* strain GV3101 harboring the various combinations of constructs was infiltrated into the abaxial sides of 3-week-old tobacco plants. Subsequently, epidermal cells of infiltrated tobacco leaves were examined for fluorescence using the confocal laser scanning microscope. The detailed procedure has been previously reported (Ok et al., 2015).

### Purification of glutathione *S*-transferase (GST) fusion proteins from *Escherichia coli*

GST-fusion proteins such as GST-CBL4, GST-CBL10-cMyc, and GST-TOC34 were purified basically according to the protocols described earlier (Ok et al., 2015). Briefly, *E. coli* BL21 cells possessing a GST fusion construct were cultured at 37°C overnight and were subcultured until the OD_600_ reached 0.5–0.6. Following 3-h induction with 0.3 mM Isopropyl-β-D-thiogalactopyranoside at 20°C, the cells were lysed in ice-cold buffer (50 mM Tris-HCl, pH 7.4, 100 mM NaCl, 1 mM PMSF, 5 mM DTT, 5 mM EDTA, and 1 mM EGTA). Glutathione-Sepharose 4B beads were used to retrieve the GST fusion protein. Ice-cold washing buffer (50 mM Tris-HCl, pH 7.4, 100 mM NaCl) was used to wash the beads. Protein concentration was determined according to Bradford (1976).

### Pull-down assay and immunoblot analysis

Pull-down assay was performed as described previously (Ok et al., 2015). Briefly, GST fusion proteins attached to the glutathione-Sepharose 4B beads were incubated at 4°C with prey proteins lacking the GST protein in the binding buffer (50 mM Tris-HCl, pH 7.4, 100 mM NaCl, 0.05% Tween 20, and 1 mM PMSF) supplemented with either 0.2 mM CaCl_2_ or 1 mM EGTA. Pull-down samples were resolved by SDS-PAGE, transferred onto polyvinylidene fluoride (PVDF) membranes (Millipore, USA), and detected by immunoblot analysis as described previously (Shi et al., 1999).

### GTPase assay

To investigate the GTPase activity of TOC34, we performed GTPase assay using a Quantichrom™ATPase/GTPase assay kit (BioAssay Systems, USA). The GTP hydrolysis activity of the TOC34 protein purified from *E. coli* was determined according to manufacturer's instructions. Briefly, the assays were initiated by adding 10 μL of TOC34 (2.5 μg) into 30 μL reaction samples containing 20 mM Tris (pH 7.0), 40 mM NaCl, 4 mM MgAc_2_, 0.5 mM EDTA, 4 mM GTP, and 5 mM CaCl_2_ or 2 mM EGTA. Depending on reaction conditions, either 2.1 μg of CBL10 or 1.8 μg of CBL4 was also included. After incubation at 27°C for 30 min, free phosphates generated were quantified according to the manufacturer's protocols using a VersaMax ELISA microplate reader (Molecular Devices, USA).

### Construction of plasmids

The following plasmids were constructed as described previously (Halfter et al., 2000; Ok et al., 2015); pGBT·CBL1, pGBT·CBL2, pGBT·CBL3, pGBT·CBL4, pGBT·CBL5, pGBT·CBL6, pGBT·CBL7, pGBT·CBL8, pGBT·CBL9, pGBT·CBL10, pGEX·CBL4, pUC-SPYNE-bZIP63, and pUC-SPYCE-bZIP63. To make pGAD·CBL10, the coding region of the *CBL10* gene was PCR amplified with a primer set of CBL10-1/CBL10-2 using pGBT·CBL10 as template. Following digestion with *Bam*Hl/*Sal*I, the PCR product was subsequently ligated into the pGAD.GH vector. To create the pGBT·TOC34 and pGAD·TOC34 plasmids, the coding region of the *TOC34* cDNA was first amplified with primers TOC34-1 and TOC34-2. Then, the resulting PCR product was digested with *Bam*HI/*Sal*I and ligated into pGAD.GH and pGBT9.BS, respectively. The plasmid pGAD·TOC34-N was constructed by cloning the PCR product amplified with TOC34-2 and TOC34-7 primers into the *Bam*HI/*Sal*I sites of the pGAD.GH plasmid. Similarly, the pGAD·TOC34-C1 and pGAD·TOC34-C2 plasmids were generated using primer sets TOC34-8/TOC34-1 and TOC34-9/TOC34-1, respectively.

The plasmid pGEX·TOC34 was constructed by cloning the PCR product amplified with TOC34-5/TOC34-6 primer set into the *Bam*HI/*Sal*I sites of the pGEX.4T-3 plasmid (GE Healthcare Life Sciences). For construction of the pGEX·CBL10 and pGEX·CBL10-c-myc plasmids, each of the PCR products amplified with primer sets (CBL10-3/CBL10-4 and CBL10-3/cMYC-1) were cloned into the pGEX-4T-3 vector, which was digested with *Bam*HI/*Sal*I and *Bam*HI/*Not*I, respectively. For creation of the TOC34-GFP chimeric construct (pMD·TOC34), primers TOC34-3 and TOC34-4 were used to PCR amplify the *TOC34* coding region without a stop codon. Following digestion with *Xba*I/*Bam*HI, the PCR product was cloned into the pMD1 binary vector that contains a GFP reporter gene (Sheen et al., 1995). For BiFC assays in Arabidopsis protoplasts, the pUC-SPYNE-TOC34 (pUC-TOC34-YFP^N^) and pUC-SPYCE-CBL10 (pUC-CBL10-YFP^C^) plasmids were created by cloning each coding region of TOC34 and CBL10, which were PCR amplified with TOC34-6/TOC34-7 and CBL10-3/CBL10-4 primer sets, into the *Bam*HI/*Sal*I sites of the pUC-SPYNE and pUC-SPYCE vectors, respectively. In addition, the TOC34:cMyc-YFP^N^ and CBL10:HA-YFP^C^ regions were PCR amplified from the pUC-TOC34- YFP^N^ and pUC-CBL10- YFP^C^ constructs with the primer sets (TOC34-3/YFPN-1 and CBL10-5/YFPC-1, respectively) and then each of them were cloned into the *Xba*I/*Bam*HI sites of the pCAM35S and pBI121ΔGUS binary vectors, thereby creating TOC34-YFP^N^ and CBL10-YFP^C^. These two constructs were used to perform BiFC assays in tobacco plants. All the PCRs were carried out using *Pfu* DNA polymerase (Stratagene) to enhance fidelity. All the constructs above were verified by DNA sequencing.

### Oligonucleotide primers used in the plasmid construction

Primers used in this study were listed below, with restriction enzyme sites underlined. Three additional bases, which were chosen randomly by considering their effect on melting temperature and on dimer and stem-loop formation, were included at the 5′ end of the primers for efficient digestion by restriction enzymes:

TOC34-1, 5′-ATT GTC GAC ACT CAA GAC CTT CGA CTT GC-3′
TOC34-2, 5′-TAA GGA TCC CAT GGC AGC TTT GCA AAC GC-3′
TOC34-3, 5′-ATA TCT AGA ATG GCA GCT TTG CAA ACG CT-3′
TOC34-4, 5′-ATA GGA TCC AGA CCT TCG ACT TGC TAA AC-3′
TOC34-5, 5′-ATA GGA TCC ATG GCA GCT TTG CAA ACG CT-3′
TOC34-6, 5′-ATA GTC GAC AGA CCT TCG ACT TGC TAA AC-3′
TOC34-7, 5′-ATA GTC GAC TGG CCC TTC GAC CAG TTT CT-3′
TOC34-8, 5′-AAA GGA TCC CAA CCC AAA CGA AAG AGG AA-3′
TOC34-9, 5′-TTA GGA TCC CCC ATT GGT TCG AGC AAT CA-3′
YFPN-1, 5′-TTT GGA TCC GGC CAT GAT ATA GAC GTT GT-3′
YFPC-1, 5′-TTA GGA TCC CTT GTA CAG CTC GTC CAT GC-3′
CBL10-1, 5′-TAA GGA TCC CAT GAC AAC TGG CCG ACC AA-3′
CBL10-2, 5′-ATT GTC GAC TCA GTC TTC AAC CTC AGT GT-3′
CBL10-3, 5′-ATA GGA TCC ATG ACA ACT GGC CGA CCA AA-3′
CBL10-4, 5′-TAA GTC GAC GTC TTC AAC CTC AGT GTT GA-3′
CBL10-5, 5′-ATA TCT AGA ATG ACA ACT GGC CGA CCA AA-3′
cMYC-1, 5′-AAA GCG GCG CAA GAT CCT CCT CAG AAA TCA-3′

## Results

### Isolation of a novel CBL10 interactor, TOC34

To identify a novel interaction partner(s) of CBL10, we extensively screened the Arabidopsis cDNA expression libraries CD4-10 and CD4-22 obtained from the Arabidopsis Biological Resource Center via a yeast two-hybrid system using CBL10 as a bait. The CBL10 bait (pGBT·CBL10 or BD·CBL10) was made by cloning the open reading frame (ORF) of *CBL10* cDNA into the GAL4 DNA-binding domain vector pGBT9.BS (BD). We obtained a total of 38 positive clones from this screening and found out that most of them derived from the CIPK members such as CIPK6 and CIPK24, which are previously known CBL10 interactors (Kim et al., 2007; de la Torre et al., 2013). Isolation of these genes indicated that the yeast two-hybrid screening had been performed efficiently and successfully. Among the positive clones, we were also able to discover a novel gene that does not belong to the CIPK family. Sequence analysis via GenBank (http://www.ncbi.nlm.nih.gov) showed that this gene is *TOC34* (Translocon of the Outer membrane of the Chloroplasts; *At5g05000*) encoding a polypeptide with an estimated molecular mass of 34.7 kDa. It was reported to act as a GTP-dependent receptor at the chloroplast surface (Andrès et al., 2010).

**Figure 1:**
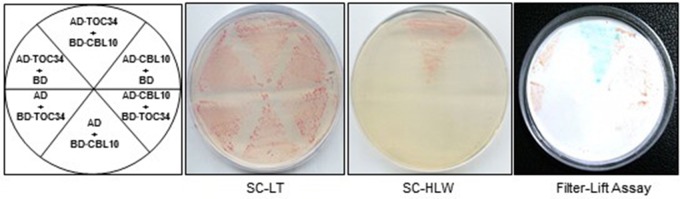
**A yeast two-hybrid assay showing that TOC34 interacts with CBL10 in a vector-dependent manner**. The first panel at left shows the arrangement of the Y109 yeast cells harboring the indicated BD and AD plasmids. The second and third panels display yeast growth on synthetic complete medium lacking Leu and Trp (SC-LW) and synthetic complete medium lacking His, Leu, and Trp (SC-HLW), respectively. The last panel shows β-galactosidase activity using filter-lift assay.

To test whether the full-length TOC34 protein interacts with CBL10 in yeast cells, we constructed the pGAD·TOC34 (AD·TOC34) plasmid by cloning the ORF of *TOC34* cDNA into the yeast expression vector pGAD·GH containing the GAL4 activation domain (AD). As shown in Figure 1, the Y190 yeast cells carrying both AD·TOC34 and BD·CBL10 grew well on the selection medium (SC-HLW) and exhibited a blue color in the filter-lift assay, indicating expression of the *HIS3* and *LacZ* reporter genes, respectively. However, a vector-swapping analysis showed that the yeast cells harboring AD·CBL10 and BD·TOC34 did not express the two reporter genes, suggesting that the interaction between TOC34 and CBL10 occurs in a vector-dependent manner. Meanwhile, the yeast cells cotransformed with the empty vectors (either with BD·TOC34 and AD, or with BD and AD·TOC34), which were used as negative controls, failed to express the reporter genes. Together, these results clearly indicated that TOC34 interacts with CBL10 in the yeast two-hybrid system.

### TOC34 interacts with cbl10 and cbl7 in the yeast two-hybrid system

**Figure 2:**
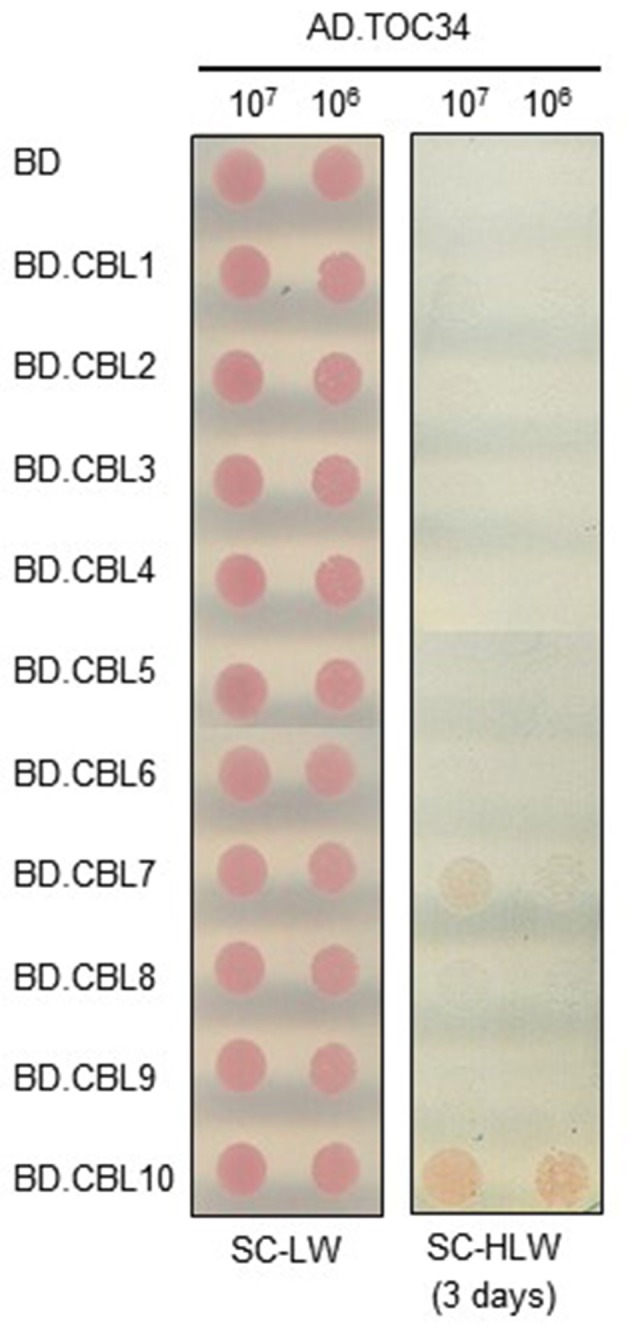
**Interaction specificity of TOC34 with CBLs in a yeast two-hybrid assay**. Yeast cells (Y190 strain) were cotransformed with the combinations of the BD and AD constructs as indicated and selected on the synthetic complete media lacking Leu and Trp (SC-LW). Cotransformed yeast cells were cultured, serially diluted, and spotted onto the indicated media. Yeast growth on the synthetic complete media lacking His, Leu, and Trp (SC-HLW) indicates interaction.

Because the CBL family members consist of 10 genes in Arabidopsis genome (Luan et al., 2002; Kolukisaoglu et al., 2004), we also investigated whether or not the TOC34 protein could interact with other members of the CBL family using the yeast two-hybrid system. To do this, we first cloned each of the 10 CBL ORFs into the BD vector (Ok et al., 2015) and then introduced them into the Y190 yeast cells harboring AD·TOC34. As shown in Figure 2, TOC34 was able to also interact with CBL7 besides CBL10, but not with other CBL family members. However, it should be noted that TOC34 interacted with CBL7 at much lower strength than with CBL10, indicating that TOC34 possesses different binding interaction affinities toward the two CBL members. This finding strongly suggests that each CBL family member can have distinct interacting partners in addition to the previously reported targets, CIPK and AtMTAN proteins (Oh et al., 2008; Luan, 2009; Ok et al., 2015).

### The C-terminal region of TOC34 is required for interaction with cbl10

**Figure 3:**
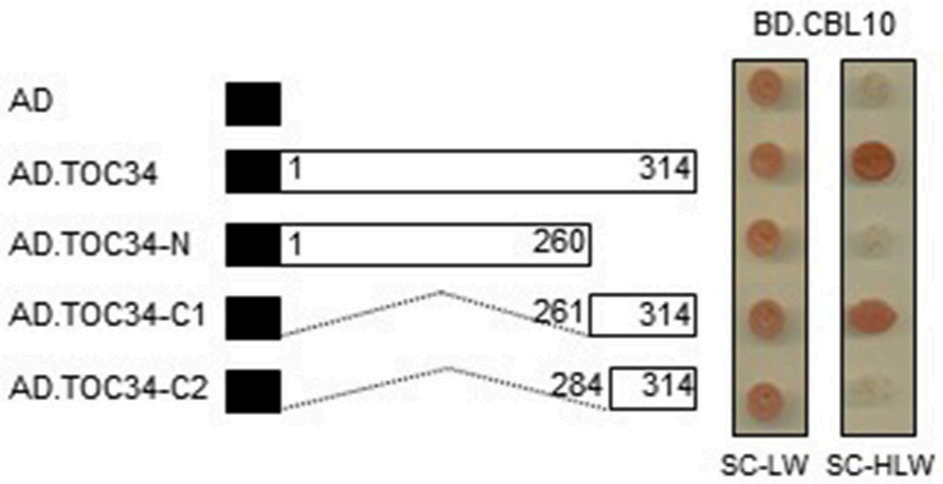
**Identification of the TOC34 region responsible for the interaction with CBL10**. A full and two deletion constructs of TOC34 were cloned into the pGAD vector and cotransformed into yeast cells with pGBT.CBL10. Yeast growth on the synthetic complete media lacking His, Leu, and Trp (SC-HLW) indicates interaction. Numbers in the white boxes indicate the beginning and the ending positions of each protein fragment. Black boxes denote the activation domain of the GAL4 transcription factor. Numbers indicate the beginning and the ending positions of each protein fragment.

To delimit the TOC34 region necessary for the interaction with CBL10, we created a series of deletion constructs by cloning TOC34 fragments into the AD vector. These constructs were then introduced into the Y190 yeast cells carrying either BD or BD·CBL10. Interactions were determined by monitoring whether or not the transformed yeast cells grow well on the selection medium (SC-HLW). As shown in Figure 3, removal of the last 54 amino acids from the C-terminal end of TOC34 (AD·TOC34-N) completely disrupted its interaction with CBL10, whereas the N-terminal deletion down to 260th amino acid residue containing small GTP-binding domain of TOC34 (AD·TOC34-C1) maintained the interaction with CBL10. However, a further deletion down to 283th amino acid residue (AD·TOC34-C2) containing a transmembrane domain (267 to 283 amino acid residues) resulted in abolishing the interaction. These results indicated that the 23-amino acid residues of TOC34, spanning from 261 to 283, are critical and necessary for the interaction with CBL10.

### CBL10 forms a complex with TOC34 *in vitro*

**Figure 4:**
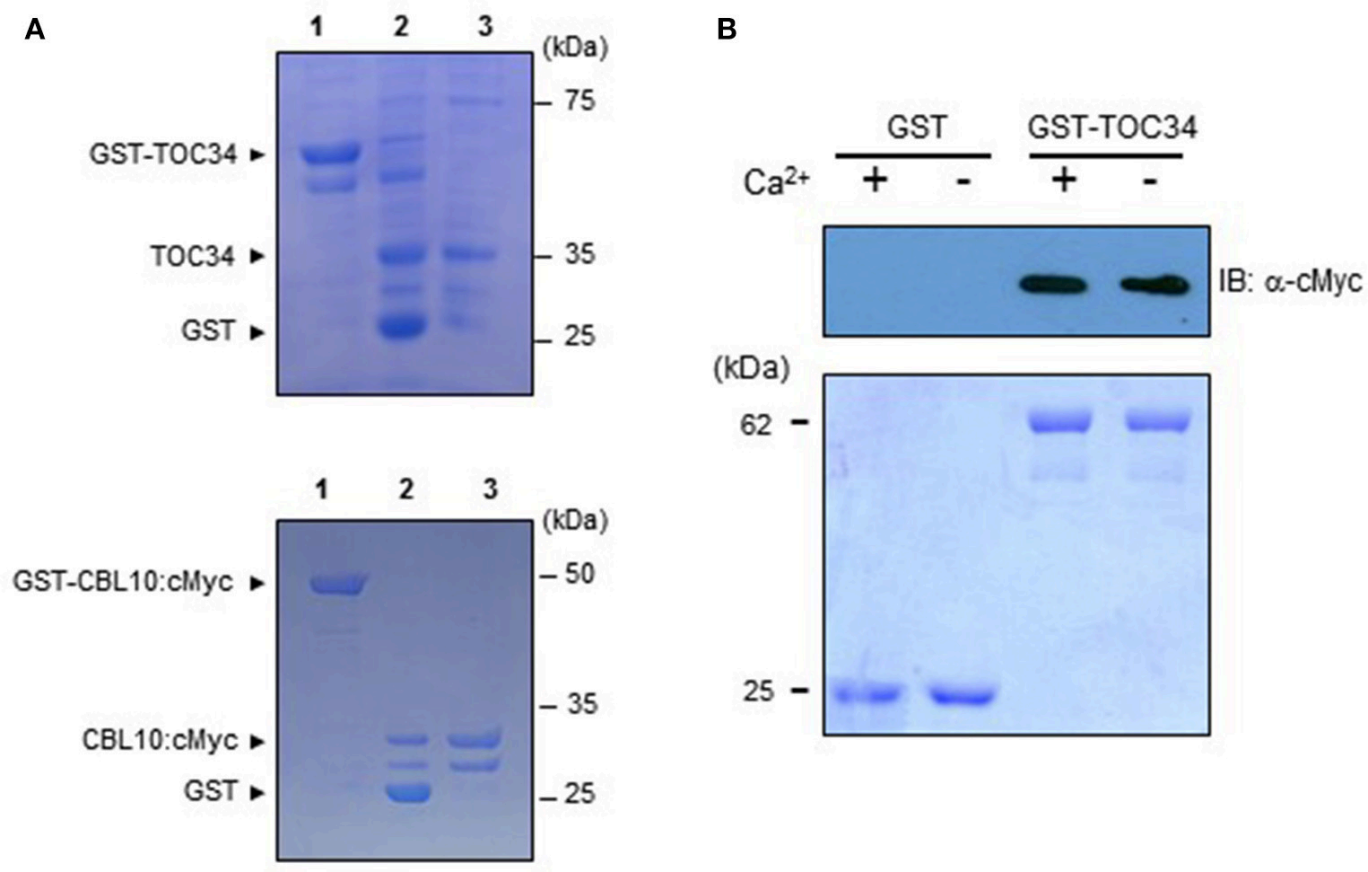
**Interaction between TOC34 and CBL10 in a calcium-independent manner. (A)** Purification of the recombinant TOC34 (top) and CBL10:cMyc (bottom) proteins expressed in *Escherichia coli*. Lanes 1 to 3 contain the GST-TOC34 or CBL10:cMyc, the thrombin-digested forms of GST and TOC34 or CBL10:cMyc, and purified TOC34 or CBL10:cMyc, respectively. SDS-PAGE gels were stained with Coomassie Brilliant Blue. **(B)** Pull-down assy. The GST-TOC34 fusion protein was used as a bait to pull down the prey CBL10:cMyc in the presence (+, 1mM CaCl_2_) or absence (−, 2mM EGTA) of calcium. For a negative control, GST alone was used as a bait. The top panel shows a western blot probed with rabbit anti-cMyc antibody and the bottom panel shows a Coomassie Brillant Blue stained SDS-PAGE gel indicating the amount of bait proteins used in each pull-down assay.

We performed pull-down assays in order to verify *in vitro* the CBL10-TOC34 interaction demonstrated in the yeast two-hybrid system (Figures 1, 2). To do this, we expressed and purified both TOC34 and CBL10-cMyc proteins from *E. coli* using the glutathione *S*-transferase (GST) gene fusion system. Figure 4A shows an approximately 35 kDa TOC34 protein band that was originally purified as a GST-fusion form and subsequently digested with thrombin to remove the GST protein. In the same way, the CBL10-cMyc protein was also prepared (Figure 4A). For the pull-down assays, we incubated GST-TOC34 (bait) with CBL10-cMyc (prey) in the presence or absence of Ca^2+^ and examined whether GST beads pulled down the CBL10-cMyc prey protein with the immunoblot analyses using anti-cMyc antibody as a probe. As shown in Figure 4B, the GST-TOC34 bait protein successfully retrieved CBL10-cMyc in the presence and absence of Ca^2+^, indicating that Ca^2+^ does not have significant influence at least on the interaction strength between CBL10 and TOC34. In contrast, GST protein alone used as a negative control failed to pull down CBL10-cMyc in both conditions. These *in vitro* protein-protein interaction assays demonstrated that CBL10 and TOC34 physically interact with each other regardless of Ca^2+^.

### CBL10 and TOC34 associate with each other in plant cells

**Figure 5:**
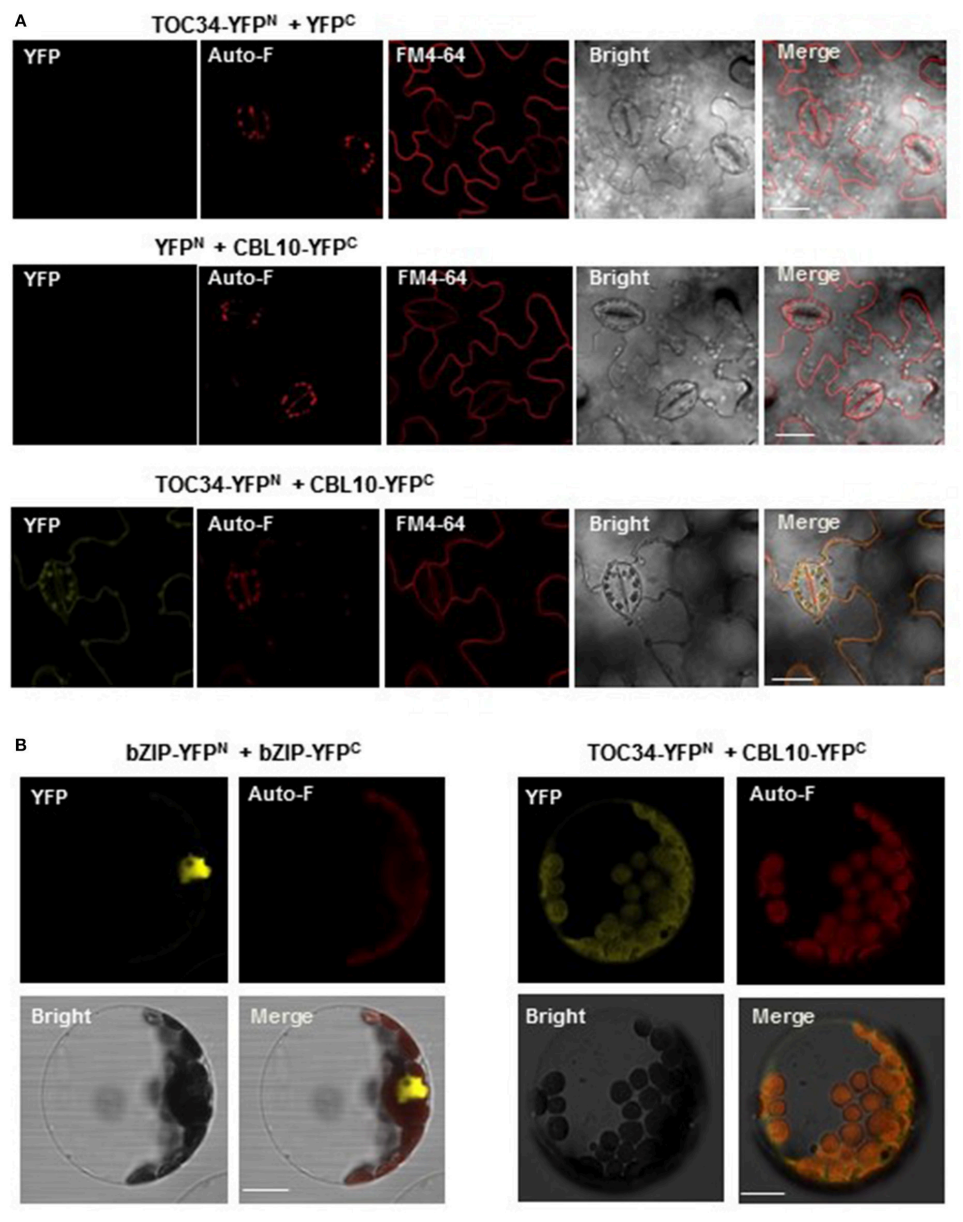
*****In vivo*** interaction between CBL10 and TOC34. (A)** Bimolecular fluorescence complementation (BiFC) analysis in tobacco (*Nicotiana benthamiana*) leaf epidermal cells infiltrated with Agrobacterium carrying indicated plasmid sets; TOC34-YFP^N^ + YFP^C^ (top row), YFP^N^ + CBL10-YFP^C^ (middle row), and TOC34-YFP^N^ + CBL10-YFP^C^ (bottom row). YFP, Auto-F, FM4-64, Bright, and Merge indicate YFP fluorescence signal, auto-fluorescence signal, N-(3-triethylammoniumpropyl)-4-(p-diethylaminophenyl-hexatrienyl) pyridinium dibromide (as a membrane marker) staining signal, bright field image, and merged image, respectively. *Scale bars*, 40 μm. **(B)** BiFC analysis in Arabidopsis protoplasts expressing bZIP-YFP^N^ and bZIP-YFP^C^ (left two columns) or TOC34-YFP^N^ and CBL10-YFP^C^ (right two columns). *Scale bars*, 20 μm.

We also carried out the bimolecular fluorescence complementation (BiFC) assay to further confirm the CBL10-TOC34 interaction *in vivo* (Walter et al., 2004). To do this, we first created TOC34-YFP^N^ and CBL10-YFP^C^ chimeric constructs by fusing TOC34 and CBL10 to the N-terminal YFP fragment (YFP^N^) and the C-terminal YFP fragment (YFP^C^) in the binary vectors, respectively. Subsequently, these constructs were transiently expressed in the tobacco leaves via an *Agrobacterium*-infiltration method. As shown in the Figure 5A fluorescence images, YFP signals were observed strongly at the outer membrane of chloroplasts and weakly at the plasma membrane of the transformed tobacco cells, indicating a physical association between CBL10 and TOC34 in a living tobacco leaf cell. The fluorescent marker dye FM4-64 was used to visualize the plasma membrane. However, no yellow fluorescence was observed from the tobacco cells expressing either TOC34-YFP^N^/YFP^C^ or YFP^N^/CBL10-YFP^C^ (a negative control).

Furthermore, we also verified the CBL10-TOC34 interaction in Arabidopsis protoplasts transfected with pUC-TOC34-YFP^N^ and pUC-CBL10-YFP^C^ chimeric constructs. Similar to the result obtained from the tobacco leaf cells, strong YFP signals were detected around the chloroplast (Figure 5B). The basic leucine zipper (bZIP) transcription factor 63, bZIP63 (At5g28770), was used as a positive control, because it is known to form a homodimer in the nucleus (Sibéril et al., 2001). Taken together, these results clearly indicated that the physical interaction of CBL10 with TOC34 occurs primarily at the chloroplast *in vivo*.

### Expression patterns of *TOC34* and subcellular localization of TOC34

**Figure 6:**
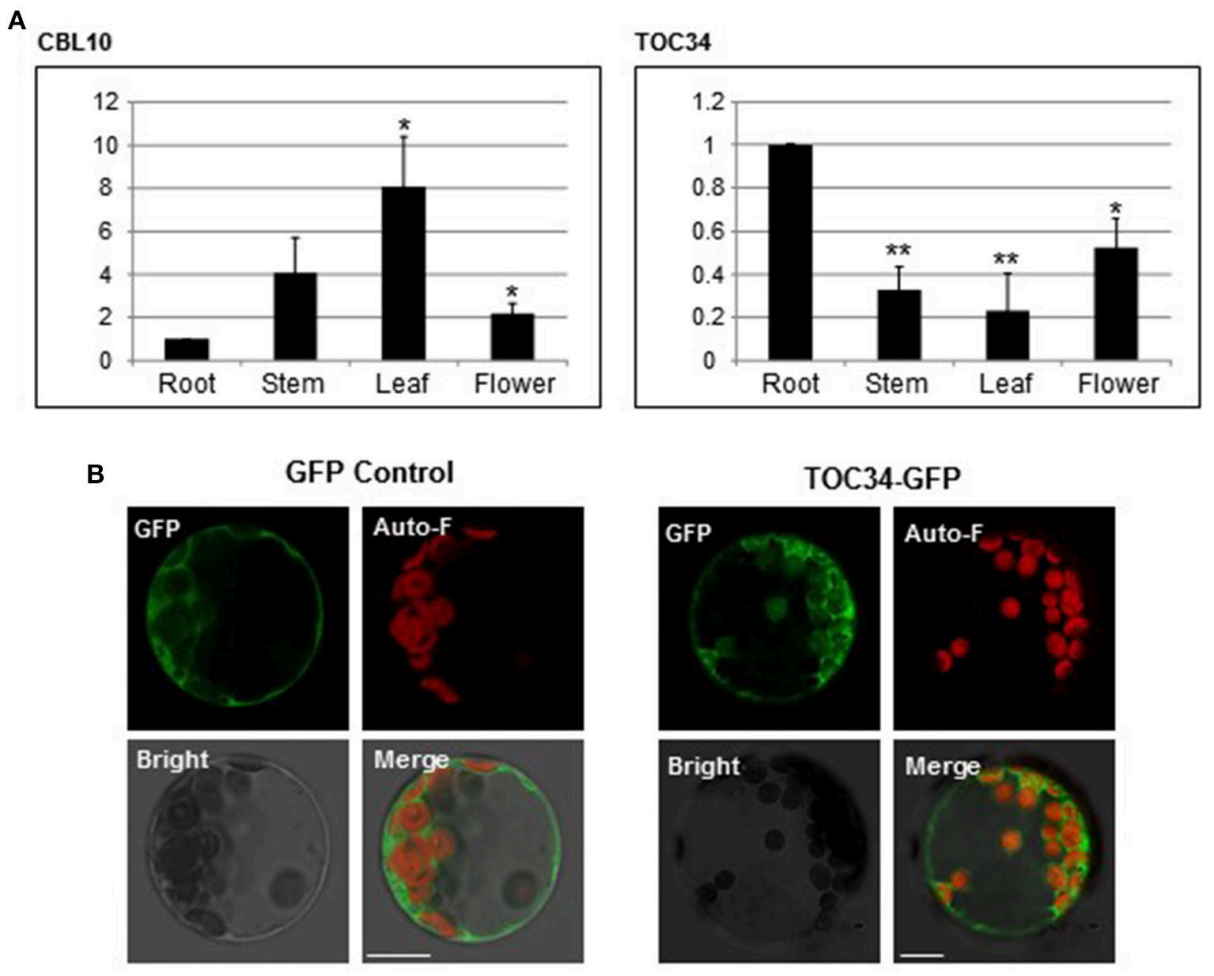
**Expression patterns of ***TOC34*** and subcellular localization of TOC34. (A)** Expression patterns of *TOC34* and *CBL10* using qRT-PCR analysis. The expression levels of *TOC34* and *CBL10* in roots were set to 1.0 to show relative abundance differences. Actin2 gene was employed as an internal control. Error bars denote standard deviation (SD) of three technical replicates. Significance was calculated using the Student's *t*-test: ^*^P < 0.05, ^**^P < 0.01. **(B)** Localization pattern of TOC34. Arabidopsis protoplasts were transfected with *35S::GFP* (left two columns) or *35S::TOC3-GFP* (right two columns), and GFP fluorescence was examined. GFP, Auto-F, Bright, and Merge indicate GFP fluorescence signal, auto-fluorescence signal, bright field image, and merged image, respectively. *Scale bars* 15 μm (left two columns) and 20 μm (right two columns).

Our results above clearly indicated that CBL10 associates with TOC34 when they exist in the same space. Therefore, it is very important to investigate the spatial expression patterns of the CBL10 and TOC34 genes in order to determine whether the CBL10-TOC34 association indeed occurs in Arabidopsis plant cells. We performed qRT–PCR analysis using total RNA prepared from the various organs of 6-week-old Arabidopsis plants [ecotype Columbia (Col-0)], which include leaves, stems, flowers, and roots. As shown in Figure 6A, both TOC34 and CBL10 genes were broadly and significantly expressed in the vegetative and reproductive organs with some differences in their expression patterns and levels. *TOC34* transcripts were most abundantly expressed in roots, and to a lesser extent, in flowers, stems, and leaves, which coincides with the published result (Gutensohn et al., 2004). In the case of *CBL10*, however, the highest expression level was observed in the leaves although the other organs also accumulated the transcripts at fairly high levels. This was also in good agreement with the previous findings (Kim et al., 2007; Quan et al., 2007). It should be noted that *CBL10* was more strongly expressed in all organs tested in this study than *TOC34*. Anyway, *TOC34* and *CBL10* clearly displayed overlapping spatial expression patterns in Arabidopsis plants, and therefore it seems reasonable to conclude that the two proteins actually associate with each other in Arabidopsis cells.

We further examined the subcellular localization of TOC34 in Arabidopsis cells, although our BiFC results indicated that TOC34 can be localized at the outer membrane of the chloroplasts and the plasma membrane when co-expressed with CBL10 in plant cells. To this end, we first fused GFP to the C-terminal end of TOC34 and created a TOC34-GFP chimeric gene, which is driven by the cauliflower mosaic virus *35S* promoter (pMD·TOC34). TOC34-GFP fusion protein was then transiently expressed in Arabidopsis protoplasts and visualized with confocal laser scanning microscopy. According to the fluorescence images (Figure 6B), TOC34-GFP appeared to be predominantly localized at the chloroplasts (probably, the outer envelope membrane) and to a much lesser extent at the plasma membrane, which is generally consistent with the previous report (Dhanoa et al., 2010). The GFP control, however, exhibited similar intensities of fluorescence throughout the cytoplasm.

### Ca^2+^-bound CBL10 significantly inhibits the GTPase activity of the TOC34 protein

**Figure 7:**
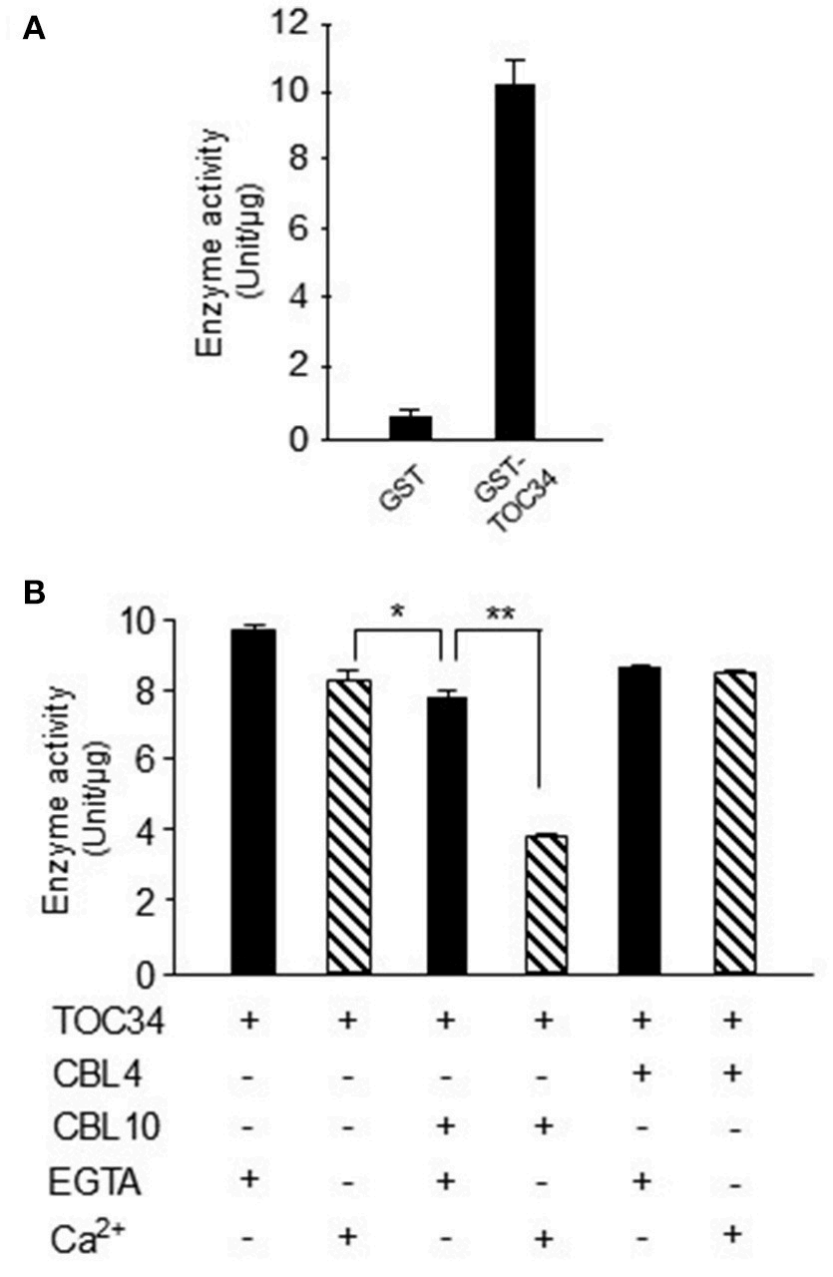
**Inhibition of GTPase activity of TOC34 by Ca^**2+**^-bound CBL10. (A)** The GTPase activity of purified GST-TOC34 protein. The GTPase activity is represented with the amount of free phosphate generated from the hydrolysis of GTP by the action of 4 μg of GST-TOC34 for 30 min at 27°C. GST protein was used as a negative control. **(B)** The effect of CBL10 or CBL4 on the GTPase activity of TOC34. The plus (+, 5 mM CaCl_2_) and minus (−, 2mM EGTA) signs indicate the presence and absence, respectively, of the indicated components in the reaction samples. Each value represents the average of three independent experiments. Error bars indicate SD. Significance was calculated using the Student's *t*-test: ^*^P < 0.05, ^**^P < 0.01.

Because TOC34 acts as a GTP-dependent receptor at the outer membrane of the chloroplast (Jarvis et al., 1998), we attempted to empirically determine whether the recombinant GST-TOC34 protein prepared from *E. coli* is able to hydrolyze GTP into GDP and inorganic phosphate. As shown in Figure 7A, GST-TOC34 successfully yielded free phosphate (~10 U/μg), whereas GST alone did not. These enzyme assays clearly suggested that the TOC34 protein indeed possesses the GTPase activity *in vitro*. Because TOC34 can form a complex with the Ca^2+^-binding protein CBL10, we also examined the effect of CBL10 on the GTPase activity of the TOC34 protein in the presence or absence of Ca^2+^. As shown in Figure 7B, the GTPase activity of TOC34 was most significantly decreased when both CBL10 and Ca^2+^ were present together. Without either Ca^2+^ or CBL10, however, such a strong inhibitory effect was not detected. Furthermore, it is also important to noted that CBL4, which is another member of the CBL family and does not associate with TOC34, did not exert any substantial effect on the TOC34 enzymatic activity regardless of Ca^2+^. Together, these results clearly indicate that the GTPase activity of TOC34 can be specifically inhibited by the association with Ca^2+^-bound CBL10.

## Discussion

The CBL family represents a unique group of Ca^2+^-binding proteins in plants and consists of 10 genes in Arabidopsis and rice (Kolukisaoglu et al., 2004). Researches with Arabidopsis CBL members have contributed greatly to our understanding of the plant Ca^2+^ signaling cascades mediated by CBLs. Currently, we are well aware of that CBLs activate the enzymatic activity of CIPKs in the presence of Ca^2+^ triggered by a variety environmental stimuli such as cold, high salinity, high Mg^2+^ concentration, low levels of nutrients such as potassium and nitrate, high pH, and osmotic stress (reviewed in Kim, 2013; Léran et al., 2015; Tang et al., 2015). Perhaps the best known example is the SOS (salt overly sensitive) signaling pathway constituted of SOS3, SOS2, and SOS1, which provides a good molecular mechanism how a CBL–CIPK complex mediates the salinity-induced Ca^2+^ signal and confers salt tolerance to plants. The genomic loci *SOS3* and *SOS2* actually encode the CBL4 and CIPK24 proteins, respectively. Sensing the Ca^2+^ signal elicited by salt stress, CBL4/SOS3 enables CIPK24/SOS2 to phosphorylate and activate the plasma membrane-bound Na^+^/H^+^ antiporter encoded by SOS1. The activated SOS1 then removes excess Na^+^ from the cytosol of plant cells, resulting in tolerance to high salinity conditions (Qiu et al., 2002; Quintero et al., 2002).

CBL10 is also known to play a role in salt tolerance by associating with and activating CIPK24/SOS2 (Kim et al., 2007; Quan et al., 2007). Unlike CBL4/SOS3 that functions almost exclusively in the roots, however, CBL10 mainly works in the shoots and leaves by recruiting CIPK24/SOS2 to the vacuolar membrane (tonoplast). The knock-out Arabidopsis mutant lacking the CBL10 activity (*cbl10*) showed the salt-sensitive phenotype and accumulated much less Na^+^ than the wild type under either normal or high salt conditions, suggesting that CBL10 is necessary for the sequestration of cytosolic Na^+^ into the vacuole (Kim et al., 2007). It is believed that CIPK24/SOS2 recruited to the tonoplast by CBL10 may phosphorylate and activate an as-yet unidentified Na^+^ channel or transporter, which transports the cytosolic Na^+^ into the vacuole.

In the present work, we have identified TOC34 as a novel interaction partner protein of CBL10 and demonstrated that it possesses the GTPase activity which can be inhibited by Ca^2+^-bound CBL10. This finding, adding an additional layer of complexity on the existing CBL-mediated Ca^2+^ signaling pathways, clearly indicates that the CBL family can relay Ca^2+^ signals in more complicated ways than currently known.

### CBLs display target diversity like other Ca^2+^ sensor relays, cams and CMLs

Our extensive yeast two-hybrid screening revealed that CBL10 can interact not only with the CIPK family members but also with TOC34. Using the pull-down assays and BiFC analyses (Figures 4B, 5), we verified that the CBL10-TOC34 interaction detected in the yeast two-hybrid system takes place *in vitro* as well as *in vivo*. Furthermore, we found that although CBL10 does not need Ca^2+^ to physically interact with TOC34, it still requires Ca^2+^ to inhibit the GTPase activity of TOC34 (Figures 4B, 7B). It is obvious that the interaction alone is not enough to modulate the TOC34 enzyme activity. This type of Ca^2+^ dependency was also observed in other CBL members: CBL2 and CBL4 interacted with CIPK14 and CIPK24, respectively, regardless of Ca^2+^, and yet Ca^2+^ binding was essential for the CBL members to activate their target kinases (Halfter et al., 2000; Ishitani et al., 2000; Akaboshi et al., 2008). Crystal structure analyses of CBL4 and CIPK24 provided a useful insight into the molecular mechanism underlying CBL4-mediated activation of CIPK24 (Sánchez-Barrena et al., 2005, 2007). Upon Ca^2+^ binding, CBL4 undergoes a conformational change to trigger structural alteration in the interacting partner CIPK24, resulting in activation of the kinase activity. Therefore, it is likely that Ca^2+^-bound CBL10 also exerts its inhibitory effect on TOC34 in a way similar to the CBL4-CIPK23 case. However, further investigation is required to unravel the molecular mechanism by which CBL10 decreases the GTPase activity of TOC34 in a Ca^2+^-dependent manner.

In order for the CBL10-TOC34 association to occur in Arabidopsis plant cells, it is prerequisite for both proteins to be present in the same space. Therefore, we examined whether CBL10 and TOC34 display some overlaps in their expression patterns and subcellular localizations. First, our qRT-PCR analysis (Figure 6A) along with the previous works (Gutensohn et al., 2004; Kim et al., 2007; Quan et al., 2007) clearly indicated that both CBL10 and TOC34 can exist together in the same tissues of Arabidopsis, albeit their relative amounts are significantly different. Second, TOC34 carries a transit peptide and mainly localizes to the chloroplasts (Dhanoa et al., 2010), which was further verified by our fluorescence image analysis of the TOC34-GFP fusion protein in this study (Figure 6B). Although TOC34-GFP was also detected at the plasma membrane at a lower level, it could be an artifact due to the *35S* promoter driving overexpression of the TOC34-GFP protein in Arabidopsis cell. In the case of CBL10, it was reported to mainly localize at the cell membranes such as the plasma membrane and the tonoplast (Kim et al., 2007; Quan et al., 2007). However, our recent analysis using the ChloroP server (http://www.cbs.dtu.dk/services/ChloroP) predicted that CBL10 can be also targeted to the chloroplast, because it harbors a transit peptide sequence (42 amino acid residues) in the N terminus. In this aspect, it makes sense that the BiFC assays (Figure 5) displayed the strong YFP signals around the chloroplast, where both CBL10 and TOC34 are located. Taken together, these results strongly suggest that the CBL10-TOC34 association indeed occurs *in vivo*; therefore, CBL10 can relay the cytosolic Ca^2+^ signals to the chloroplast-localized TOC34 protein in addition to the previously known CIPK family members in Arabidopsis.

We learned through GenBank search that the Arabidopsis genome possesses an additional TOC34-like gene, *TOC33* (*At1g02280*), which encodes a polypeptide similar to TOC34 (55% identity; 72% similarity). Recently, we isolated a full-length cDNA of the gene and found that TOC33 also interacts only with CBL10 but not with the other CBL members in the yeast two-hybrid system (data not shown). It seems that both members of the TOC34 family, TOC34 and TOC33, can be modulated by CBL10. These findings, together with our previous reports in which we showed that CBL3 targets the AtMTAN family, strongly suggest that each member of the CBL family can target various proteins with different biochemical properties like other Ca^2+^ sensor relays such as CaMs and CMLs. Such target diversity not only dramatically increases the level of complexity in the CBL-mediated Ca^2+^-signaling pathways but also allows CBLs to control a much wider range of cellular and physiological processes in response to a variety of Ca^2+^-eliciting stimuli in plants. In this context, it is critical to uncover novel interaction partners for other CBL members in order to fully understand the CBL-mediated signaling network in plants.

### CBL10 can act as either activator or inhibitor depending its interaction partners

As stated above, CBL10 associates with and activates CIPK24/SOS2 in a Ca^2+^-dependent manner (Kim et al., 2007; Quan et al., 2007). Contrary to this activator role, we found in the present study that CBL10 acts as inhibitor when it forms a complex with the TOC34 protein; the GTPase activity of TOC34 was significantly decreased by Ca^2+^-bound CBL10 (Figure 7B). Therefore, it appears that CBL10, like CBL3, can also serve as either activator or inhibitor depending on its interaction partner proteins (Oh et al., 2008; Ok et al., 2015). How can Ca^2+^-bound CBL10 inhibit the GTPase activity of TOC34? Although the underlying mechanism is currently unavailable and waits to be investigated, we speculate that it has something to do with the fact that TOC34 forms a homodimer, causing a 1.5-fold increase in the GTPase activity (Reddick et al., 2007; Yeh et al., 2007; Koenig et al., 2008). In this aspect, it is interesting to note that CBL10 decreases the TOC34 GTPase activity by about 0.5-fold in the presence of Ca^2+^ (Figure 7B). Based on these facts, it is conceivable that Ca^2+^-bound CBL10 may disrupt or destabilizing the TOC34 homodimer, which is crucial for the optimal enzyme activity.

### Inhibition of the TOC34 GTPase activity by Ca^2+^-bound CBL10 may influence translocation of proteins into plastids in arabidopsis

A large number of chloroplast proteins are nuclear encoded and many of them are imported into the plastid through two hetero-oligomeric protein apparatuses, the TOC and TIC complexes (Translocon at the Outer/Inner envelope Chloroplasts) (reviewed in Oreb et al., 2008; Schleiff and Becker, 2011). TOC34 is one of the core components constituting the TOC complex and plays a critical role in the protein import process by recognizing plastid-destined precursor proteins and presenting them to the translocation channel TOC75 in cooperation with TOC159. Because the GTPase activity of TOC34 is tightly linked to the translocation efficiency, modulation of the TOC34 enzymatic activity is considered a major regulatory step in the translocation process (Paila et al., 2015). In addition, it has been known that the import of a certain subset of chloroplast proteins is regulated by Ca^2+^ (Chigri et al., 2005). In this context, our present finding may provide a novel insight into a molecular mechanism how Ca^2+^-signals elicited by environmental stresses modulate the TOC34 GTPase activity and thereby regulate protein import into the chloroplast. The physical interaction between CBL10 and TOC34 in the presence of Ca^2+^ could be the actual regulatory mechanism occurring in plants to control the activity of the TOC complex. Anyway, considering the important roles of chloroplasts in photosynthesis and other metabolic pathways in plant cells, it is not surprising that this organelle should be integrated into the Ca^2+^-signaling network and be regulated to accommodate environmental changes. Since the CBL10 and TOC34 genes are expressed in both green and non-green tissues of Arabidopsis (Figure 6A; Gutensohn et al., 2004; Kim et al., 2007; Quan et al., 2007), it appears that the CBL10-TOC34 complex functions not only in the chloroplast but also in other plastid types.

## Author contributions

KK conceived and designed the research. JC, YP, and MC conducted the experiments. KK analyzed the data. JL and KK wrote the manuscript with contributions from JC. All authors read and approved the final manuscript.

## Funding

This research was supported by Bio-industry Technology Development Program (No. 312033-5), Ministry of Agriculture, Food and Rural Affairs, South Korea. This work was also supported by the Next-Generation BioGreen21 Program (No. PJ011857012016), Rural Development Administration, South Korea.

### Conflict of interest statement

The authors declare that the research was conducted in the absence of any commercial or financial relationships that could be construed as a potential conflict of interest.
